# Editorial: The novel insight into managements of undiagnosed pleural effusion

**DOI:** 10.3389/fmed.2026.1775423

**Published:** 2026-01-23

**Authors:** Benjamin Hargreaves, Sanjeevan Muruganandan

**Affiliations:** 1 Department of Respiratory and Sleep Medicine, Northern Health, Melbourne, VIC, Australia; 2 Faculty of Medicine, Dentistry and Health Sciences, University of Melbourne, Melbourne VIC, Australia

**Keywords:** malignant pleural effusion, pleura, pleural disease, pleural fluid, pleural tuberculosis

Pleural effusions occur due to increased fluid production within, or reduced fluid resorption from, the pleural space. There are over 60 recognized causes of pleural effusions and can be related directly to the lung and pleura, or as a result of dysfunction in another organ system (1). Globally, pleural disease affects approximately 3.6 in every 1,000 people and, in America, contributes an estimated healthcare cost of $10.1 billion per year (2). Despite being a commonly encountered medical condition, the underlying cause can be elusive. The cornerstone of diagnosis, historically, has been thoracentesis with pleural fluid analysis for cytology, microbiology and biochemical criteria to apply Light's criteria. The goal of this Research Topic is to collect novel insights into the diagnostic techniques and investigations of rare and undiagnosed pleural effusions.

Mycobacterium tuberculosis (TB) infection is one of the leading causes of pleural effusions in endemic areas (3). Challengingly, rates of detection by pleural fluid staining and culture are low. Further diagnostic testing to accurately identify tuberculosis involves obtaining histologic samples via pleural biopsy, whether imaging guided or thoracoscopically. Obtaining these samples is invasive and relies on appropriate expertise. In these situations, biomarkers which can be used to infer the likelihood of infection with tuberculosis will allow the treating physician to delineate which patient would require more invasive procedures to confirm the diagnosis of pleural TB. Liu et al. developed and validated a diagnostic prediction model using seven different commonly available clinical and biochemical parameters, with better diagnostic accuracy compared to any single variable or other prediction models currently available. Diagnostic models highlight the advances in research in the field of pleural effusion but as of yet, have not surpassed histological samples for diagnostic accuracy.

Another cause of pleural effusion that is often associated with tuberculosis is those caused by constrictive pericarditis. Forty-four percent of cases of constrictive pericarditis present with pleural effusions, however these are typically within the left hemithorax (4). Wei et al. conducted a retrospective analysis focused on the investigation and workup of 19 patients with histologically confirmed constrictive pericarditis that had an initial clinical presentation of bilateral effusions. Interestingly, in 26% of the patients, tuberculosis was found to be the causative factor when examining pericardial tissue after having negative pleural fluid stains and cultures. The analysis culminated in the development of a diagnostic workup tool for effusions thought secondary to constrictive pericarditis, with a focus on combining clinical and imaging findings for higher diagnostic confidence (Wei et al.).

Malignancy is an important cause of pleural effusions. Depending on the primary malignancy, the sensitivity of pleural fluid cytology can range from 5% to 95% (5). Due to the uncertainty of making a diagnosis, some clinicians utilize biomarkers to predict the likelihood of malignancy being present. Carcinoembryonic antigen (CEA) is one of the most widely studied, with a sensitivity of 54% and specificity of 94% for diagnosing MPE (6). It has been shown that CEA levels differ between men and women, and that levels of CEA increase with age. Furthermore, recent studies indicate that other pleural fluid biomarkers such NTpro-BNP and adenosine deaminase (ADA) have decreased diagnostic accuracy with increased age (7, 8). Given this, Yang et al., performed a *post-hoc* analysis of two studies which included 210 prospective and 235 retrospective patients, to examine the effects of sex and age on CEA diagnostic accuracy (8). Sex had no effect, however age above the chosen cut-off of 55 resulted in decreased accuracy in detection of malignant effusion (Yang et al.).

Secondary pleural malignancy and effusion most commonly arise from lung and breast cancers, while genitourinary, gastrointestinal and lymphoid cancers can also be causes, though they are less frequent. The combination of an uncommon primary cancer and a low sensitivity for diagnosis on cytology provides a significant diagnostic challenge. Lu et al. describes a case in a 67-year-old woman presenting with a pleural effusion who was ultimately diagnosed with B-lymphoblastic lymphoma. Throughout, it was highlighted the importance of a thorough workup and that, despite a negative cytology, important to continue searching for the underlying cause. Ouattara et al. also presents a rare case of the hematological malignancy, Erdheim–Chester disease, manifesting as recurrent pleural effusions in a 75-year-old male. In this scenario it was the combination of pleural biopsy and a typical radiological sign of a “coated aorta” that secured the diagnosis. These cases demonstrate the necessity of a comprehensive workup for all undiagnosed pleural effusions, to not miss an essential diagnosis such as these rare malignancies.

There is much interest into what causes effusions, yet equally significant research is occurring to identify why these effusions occur. It has been suggested that the pleural space has its own unique immune identity, possibly due to the cytokine and chemokine producing capabilities of mesothelial cells (9). Flogel et al. conducted a novel prospective study comparing the immunological characteristics of paired plasma and pleural fluid samples in a cohort of children with pleural effusion post-cardiac surgery for congenital defects (Flögel et al.). This study identified significantly different immune phenotypes between the two. The pleural space was characterized by having higher levels of T-helper 1, T-helper 17 and memory effector cytotoxic T cells resulting in higher levels of the pro-inflammatory cytokines interleukin (IL)-6, IL-8 and tumor necrosis factor (Flögel et al.). If this same profile of pro-inflammatory cytokines also occurs in other diseases, it may help to explain why a wide range of conditions all lead to the same clinical manifestation of a pleural effusion.

In summary, the studies collected within this Research Topic emphasize both the progress and ongoing challenges in managing pleural effusions. The advent of novel prediction models, diagnostic pathways, ongoing evaluation of biomarkers and reviews of rare yet important cases all help to advance the field of pleural medicine. The insights and discoveries into the immunophenotype of the pleural space are in their infancy, and as they grow, seem poised to provide interesting and unique opportunities for management. Together, these advances highlight the need for ongoing novel research to further improve diagnostic accuracy and ultimately improve care for those patients who present with pleural effusions.
